# Laser therapy for treating cleft lip or/and palate scarring—a systematic review and meta-analysis

**DOI:** 10.1007/s10103-024-04082-3

**Published:** 2024-06-20

**Authors:** Yixin Sun, Ziming Li, Xiaoyu Qi, Binghan Wang, Nanze Yu, Jiuzuo Huang, Wenyun Ting, Xiao Long

**Affiliations:** Department of Plastic and Aesthetic Surgery, Peking Union Medical College Hospital, Chinese Academy of Medical Sciences and Peking Union Medical College, No. 1, Shuaifuyuan, Dongcheng District, Beijing, 100005 China

**Keywords:** Laser therapy, Scar, Cleft lip, Cleft palate

## Abstract

This review aims to assess the efficacy and safety of laser therapy in managing scars resulting from cleft lip and/or palate (CL/P) repair surgeries, as well as to determine the optimal timing for intervention. A systematic search was conducted across four databases using a predefined search strategy. Studies included were randomized controlled trials, non-randomized studies, and case series focusing on laser therapy for CL/P scars. Data extraction and analysis were performed using Revman Software. A total of two randomized controlled trials, four non-randomized studies, and three case series were included in the analysis. The fractional CO_2_ laser was the most commonly utilized type of laser. Following laser therapy, there was a significant decrease in Vancouver Scar Scale (VSS) scores by 4.05 (95% CI, 2.10–5.99). Meta-analysis revealed that laser treatment groups exhibited a significantly lower mean VSS score (1.3; 95% CI, 0.02–2.67) compared to control groups. Moreover, initiating laser therapy intervention at one month postoperatively resulted in a significantly lower VSS score compared to initiation at three months postoperatively (difference of 1.70; 95% CI, 1.33–2.08). No severe complications were reported. Laser therapy demonstrates effectiveness and safety in improving CL/P scars, with earlier intervention yielding greater benefits.

**Supplementary information:**

The online version contains supplementary material available at 10.1007/s10103-024-04082-3.

## Introduction

Cleft lip and/or palate (CL/P) represents one of the most prevalent congenital deformities globally, affecting approximately 1 in 600 live births [1]. Although surgical intervention is the cornerstone for this malformation, the resulting scars after surgery affect aesthetics and functional outcomes, which may further hamper individuals’ psychological well-being. Current treatments for improving surgical scars involve secondary surgery [2], silicone-based products [3], or botulinum toxin type A injection [4]. However, new methods and techniques with less invasiveness or better efficacy to eliminate scars are still needed.

In recent years, the clinical application of laser therapy has expanded rapidly, offering a non-invasive or minimally invasive option for treating various dermatological issues [5, 6]. Laser therapy has emerged as a promising adjunctive technique for the prevention and treatment of surgical scars [7]. Previous studies have demonstrated that laser therapy can improve tissue microcirculation through vasodilatation, angiogenesis, and rebuilding collagen fibers, thereby modulating the wound healing process and potentially improving scar quality [8, 9].

Although laser therapy has gradually gained attention in scar treatment, its specific application and safety in cleft lip and palate (CL/P) remain relatively underexplored. While preliminary studies indicate its potential advantages, a comprehensive assessment of existing literature is necessary to determine the true efficacy of laser therapy in this population. Therefore, this systematic review and meta-analysis aim to comprehensively explore the existing evidence regarding the use of laser therapy for preventing and treating CL/P scar formation.

## Method

Following the guidelines of the Preferred Reporting Items for Systematic Reviews and Meta-Analyses (PRISMA) protocol, this systematic review was duly registered with the PROSPERO database (registration number: CRD42024475312).

### Search strategy

A comprehensive search of the literature was conducted on February 22, 2024, across PubMed, Web of Science, Embase, and the Cochrane Library. The search terms included cleft lip and/or palate, scar, and laser, along with their respective synonyms.

### Eligibility criteria

Inclusion criteria included full-text, peer-reviewed, and original studies, which investigate the efficacy of laser therapy in treating postoperative scars associated with CL/P. There were no restrictions based on publication time, language, or laser type. Reviews, conference abstracts, animal experiments and duplicated data were excluded.

### Study selection

Two researchers (Y.S and Z.L) independently performed the initial screening of titles and abstracts from the identified literature. Subsequent full-text assessments were conducted on potentially relevant studies to ascertain their eligibility, and any discrepancies were resolved through mediation by a third reviewer (X.L).

### Data extraction

Independent data extraction was performed by two investigators (Y.S and Z.L) from the included studies, involving the collection of article publication details, baseline characteristics, intervention specifics, outcomes, and follow-up information. The integrated data were integrated using Microsoft Word and subsequently cross-referenced between the two reviewers. Any disparities were resolved through discussion, with potential adjudication by a third reviewer (W.T).

### Data analysis

A descriptive analysis of the studies was initially conducted. The inverse variance method was utilized to calculate the overall effect estimate. A random-effects model was applied if significant heterogeneity was detected, otherwise a fixed-effects model was utilized. Heterogeneity was evaluated using Cochran's Q and the I^2^ statistic, with I^2^ values exceeding 50% or P-values below 0.1 indicating significant heterogeneity. The differences in Vancouver Scar Scale (VSS) scores before and after the treatment were the main indicator for the treatment efficacy, the same as the differences in VSS scores between laser treatment and control group. Comparison of laser therapy performed in different postoperative time was also conducted to investigating the optimal timing of treatment. The continuous data was used with mean and standard deviation (SD), expressing with mean differences (MD) and calculated the 95% confidence intervals (CI). Publication bias was assessed using funnel plots. Comparative studies were separately analyzed. The meta-analyses were performed using Revman Software (version 5.4).

### Scar pathology

The normal progression of wound healing consists of three recognized phases: the initial inflammatory phase, occurring within 1–3 days post-injury, characterized by activation of the extrinsic clotting cascade and formation of a fibrin plug; the subsequent proliferative phase, spanning from 4 to 21 days post-injury, marked by granulation tissue formation, synthesis of collagen III and extracellular matrix, and angiogenesis; and the final remodeling phase, extending from 21 days to 1 year post-injury, involving granulation tissue remodeling, collagen I production, and immature blood vessels regression [10].

### Risk of bias assessment

Bias risk assessment for comparative studies adhered to the guidelines outlined in the Cochrane Handbook for Systematic Reviews (http://www.cochranelibrary.com/). Single-arm studies were categorized as high-risk for bias. Two reviewers (Y.S and Z.L) independently performed the assessment, with any disagreements resolved through mediation by a third reviewer (W.T).

## Result

**Fig. 1:**
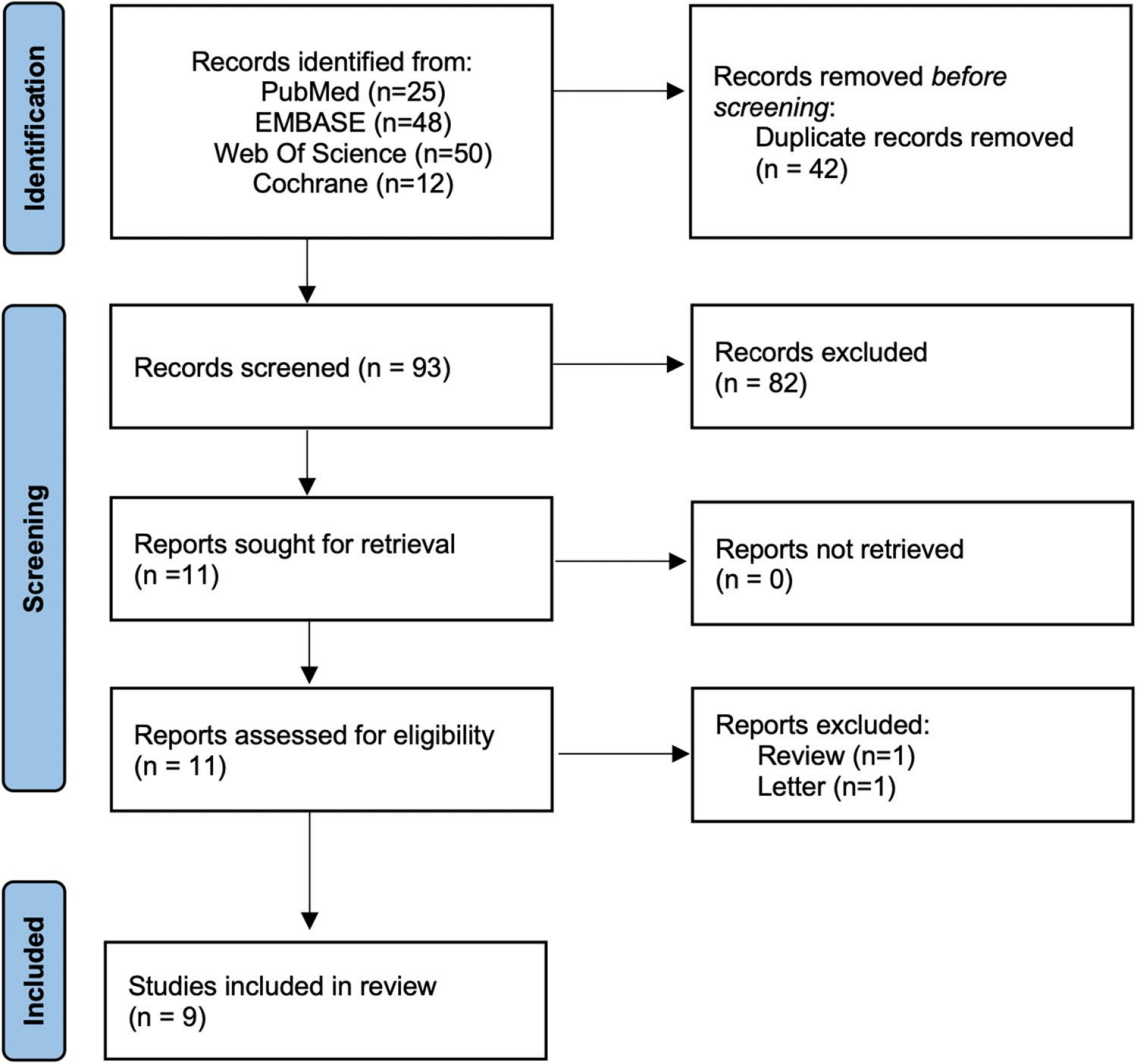
PRISMA flow chart

A total of 135 articles were retrieved from database searching. With duplicate removal, 93 articles were preliminary screened with title and abstract. Among them, eleven potentially eligible articles were identified for full-text retrieval, of which nine articles were ultimately included in the analysis. The detailed screening flow chart was presented in Fig. 1.

Among the 9 articles, there were 2 randomized controlled trials (RCTs) [8, 11], 4 non-randomized comparative studies [9, 12–14], and 3 prospective cohort [15–17] studies. Four studies were from China [8, 11, 13, 14], 3 studies from Egypt [9, 12, 17], 1 from Iran [16] and 1 from Italy [15]. This study reviewed a total of 451 patients, ranging in age from 3 months to 47 years, with a slight male predominance (58.8%). Based on available data, there were at least 25 cases of bilateral CL/P and 133 cases of unilateral CL/P. Of these, 204 patients underwent primary repair surgery, while 105 patients received secondary repair surgery. Three studies limited patients’ Fitzpatrick skin phototype to III or IV type. Detailed information for each eligible study is presented in the Table 1.

**Table 1:** Details of included studies

Study ID	Study type	Country	Patient number	Sex	Age	Type of cleft	Surgical stage	Fitzpatrick skin phototype
Chi-2024 [11]	RCT	China	18	6F 12M	6 months to 31 years	Bilateral cleft lips	4 primary 14 secondary	III, IV
Mohsen-2023 [9]	Comparative	Egypt	80	-	-	Unilateral cleft lip	Primary	-
Chi-2022 [8]	RCT	China	42	-	-	-	Secondary	-
Shadad-2021 [12]	Comparative	Egypt	120	-	-	-	-	-
Li-2019 [13]	Comparative	China	113	69M 44F	6 months to 47 years	-	43 secondary 70 primary	III, IV
Jahanbin-2019 [16]	Prospective	Iran	12	12F	Mean: 19.17 ± 3.21 years	12 cleft lip and palate	-	III, IV
Peng-2018 [14]	Comparative	China	50	31M 19F	3 months to 8 years	47 unilateral and 3 bilateral cleft lips	Primary	-
Mossaad-2018 [17]	Prospective	Egypt	6	3M 3F	Mean: 17.8 ± 2.7	-	Secondary	-
Nocini-2002 [15]	Prospective	Italy	10	-	-	Four bilateral and 6 unilateral cleft lips	-	-

*M* Male, *F* Female

**Fig. 2:**
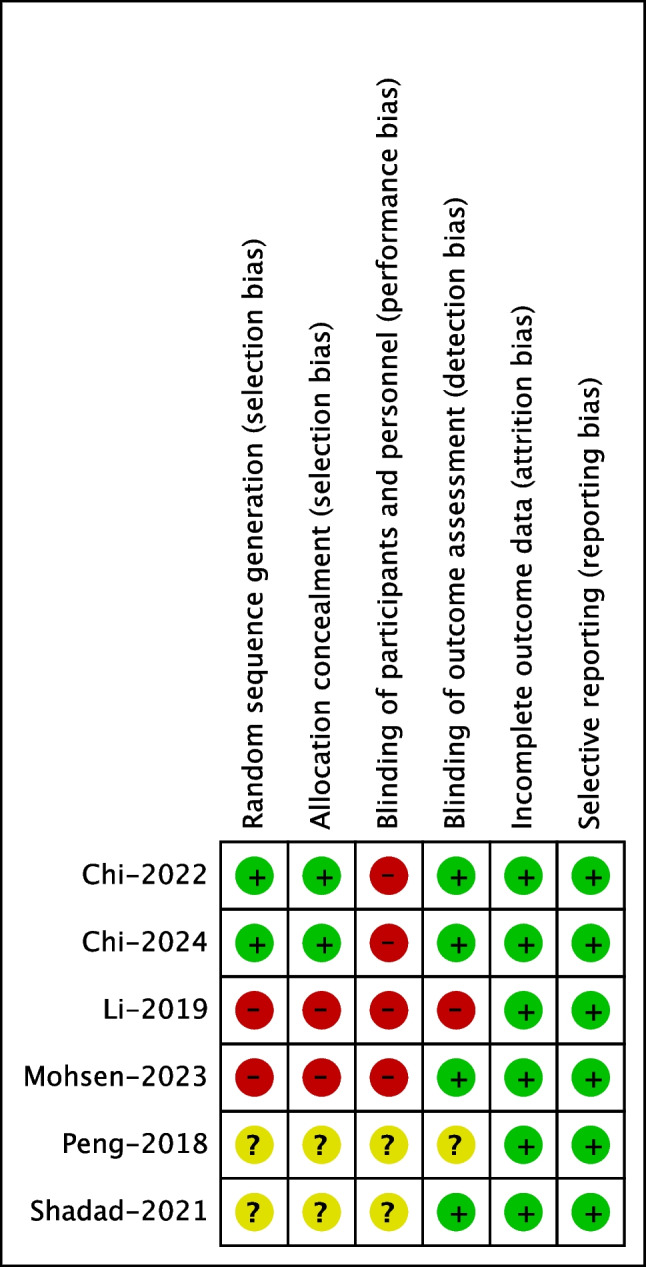
Risk of bias assessment

Two comparative studies [8, 11] exhibited a high risk of performance bias but were rated as low risk in other domains. Meanwhile, two other comparative studies [9, 13] were classified as high risk primarily due to their inadequate control over selection bias and performance bias. The remaining two comparative studies [12, 14] did not display the randomization and blinding process, thereby the relative bias risk is unclear (Fig. 2).

### Laser type

The fractional CO2 laser was the most commonly used laser type (*n* = 5), typically administered once every 4 weeks, with a total treatment course of 5–7 sessions. In a self-controlled study [11] the 595 nm pulsed dye laser (PDL) was applied every two weeks for 5 sessions. Low power diode laser 806 nm was utilized in a study with a denser interval (three times per week) and more sessions (12). Nocini et al. [15] used Er: YAG laser once every 3 months for two sessions. Peng et al. [14] combined intense pulsed laser (IPL) and fractional CO_2_ laser, administering the former once a month and the latter once every three months, resulting in a total treatment duration of six months. The intervention detail of each study was presented in the Tables 2 and 3.

**Table 2:** Detailed intervention information of comparative studies

Comparative study										
Study ID	Experimental group					Control group		Outcome Indicator	Follow-up	Complications
	population	Laser Type	Begin time of intervention	Frequency	Total sessions	population	Intervention			
Chi-2024 [11]	18	595-nm pulsed dye laser	2 week po.	Once every two weeks	5	18	None	VSS/scar area/PSAQ	9 months	1 purpura
Mohsen-2023 [9]	60	Low Power Diode Laser 806nm	1st week po.	Three times per week	12	20	None	VSS/ scar width/scar thickness	8 months	None
Chi-2022 [8]	10	Fractional CO_2_ Laser	1 month po.	Once a month	3	12	Not described	VSS	4 months	2 mild erythema
	10		3 months po.							
	10		6 months po.							
Shadad-2021 [12]	40	Fractional CO_2_ Laser	3 weeks po.	Once every 4 weeks	5–7	40	Corticosteroid creams, silicone gel	VSS/scar width/VAS	5–7 months	-
	40		3 months po.							
Li-2019 [13]	43	Fractional CO_2_ Laser	1 month to 1year po.	Monthly	6	70	None	Subjective rating scale	6 months to 5 years	-
Peng-2018 [14]	25	IPL and fractional CO_2_ laser	IPL: 2 weeks po. CO_2_ laser: 1 month po.	IPL: monthly CO_2_ laser: once every 3 months	IPL:6 CO_2_ laser: 2	25	Scar cream; silica gel massage	VSS	12 months	-

a: Po.: Postoperatively

b: VSS: the Vancouver Scar Scale

c: PSAQ: Patient Scar Assessment Questionnaire

d: VAS: Visual Analog Scale

**Table 3:** Detailed intervention information of non-comparative studies

Study ID	population	Laser Type	Begin time of intervention	Frequency	Total sessions	Outcome Indicator	Follow-up	Complications
Jahanbin-2019 [16]	12	Fractional CO_2_ Laser	Not described	Once every 4 weeks	5	Quartile grading scale/PSAQ	6 months	-
Mossaad-2018 [17]	6	Fractional CO_2_ Laser	Not described	Once every 4 weeks	6	VSS		Crust formation, pain
Nocini-2002 [15]	10	Er:YAG Laser	Not described	Once every 3 months	2	Patient satisfaction questionnaire	-	10 erythema, swelling

a: VSS: the Vancouver Scar Scale

b: PSAQ: Patient Scar Assessment Questionnaire

### VSS

**Fig. 3:**
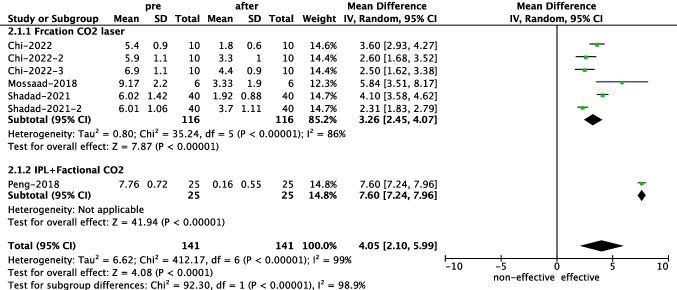
Forest plot of VSS scores

Six articles recorded the VSS scores change after laser treatment, with only four of them [8, 12, 14, 17] reporting mean values and standard deviations, which were suitable for data integration. All studies observed a significant decrease in scores after laser therapy, with an overall mean difference of 4.05 (95% CI, 2.10–5.99) (Fig. 3). The overall heterogeneity was significant with a I^2^ of 99%. The funnel plot displayed an asymmetric distribution, suggesting some publication bias (Supplemental Fig. 1). Further subgroup analysis according to the laser types showed that for factional CO_2_ laser treatment, the mean difference is 3.36 (95% CI, 2.45–4.07). One study [14] combined IPL and fractional CO_2_ laser and reported a difference of 7.60 (95% CI, 7.24–7.96).

**Fig. 4:**
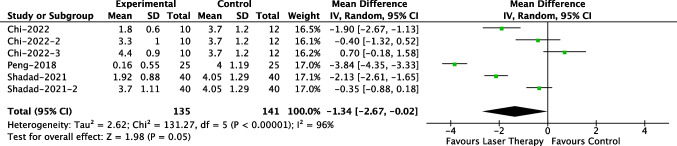
Forest plot of VSS scores for comparative studies

We also conducted separate meta-analysis for comparative results. One study [8] set three experimental groups with different intervention initiation time, while another study [12] set two experimental groups. Compared to control group (2 studies [12, 14] used scar creams and silica gel, 1 studies [8] did not provide detailed descriptions), the laser treatment groups have a lower mean VSS score of 1.34 (95% CI, 0.02–2.67) (Fig. 4).

Two studies did not report data in the form of mean and SD. One study [11] conducted a randomized, self-controlled trial aimed at evaluating the efficacy of 595-nm PDL. At the 6-month follow-up, the relative change in VSS was 0.116 ± 0.336, significantly lower than that in the control group. An open-label study [9] compared low-power 806-nm diode laser with the control group. The median VSS score of laser group (median = 3) was significantly lower than the control group (median = 6.5).

### Scar width/area/thickness

Two studies assessed the scar width. Shadad et al. [12] found that the scar width in the early intervention group (2.51 ± 0.64) was significantly lower than that in the late intervention group (3.17 ± 0.54) and the control group (3.27 ± 0.48). However, in Mohsen's study, there was no significant difference in scar width between the low-power diode laser treatment group and the control group [9]. Mohsen et al. [9] also utilized ultrasound to measure the scar thickness and observed a reduction in scar thickness on the 14th day of laser treatment [9]. Detailed data on scar width or thickness were not provided in this study.

In Chi's self-controlled study, a 3dMD photographic measurement system was used to calculate scar area [11]. The mean area on the laser side (24.20 ± 10.95 mm^2^) was significantly smaller than that on the control side (31.19 ± 14.51 mm^2^) after treatment.

### Other indicators

Li et al. [13] utilized a subjective rating scale, grading the efficacy to obviously effective, effective and ineffective according to the change of pliability, color and thickness. In the laser treatment group, there was a notable prevalence of patients exhibiting obviously effective or effective efficacy compared to those in the control group. Quartile grading scale was employed in Jahanbin’s study [16] to assess the improvement of scar texture. 0 means minimal to no improvement while 3 represents near total improvement. The results were evaluated by two blind dermatologists and showed a mean of 1.29 ± 0.86, a median of 1.25 ± 1.38 after treatment. Nocini et al. [15] used patient satisfaction questionnaire as the primary indicator and the average satisfaction level is 8.8 (1: bad;10: high).

Some adjuvant assessments also performed in some studies as efficacy indicators, such as visual analog scale (VAS) [12], patient scar assessment questionnaire (PSAQ) [11, 16]. All the relative results support the efficacy of laser therapy in improving CL/P scar.

### Optimal begin time of laser therapy

**Fig. 5:**
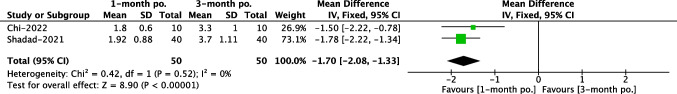
Forest plot of VSS scores comparing different initiation time of treatment

Three cohorts [9, 11, 12] started intervention at the proliferative phase and five cohorts [8, 12, 13] initiated treatment at the remodeling phase. All cohorts that commenced intervention during the proliferative phase exhibited a significant therapeutic effect. Two cohorts [8] that initiated treatment during the proliferative phase (one at 3 months and the other at 6 months after surgery) demonstrated similar improvement post-treatment compared to the control group. The resting cohorts showed significant improvement. A meta-analysis was performed to assess the efficacy of intervention initiation approximately one month postoperatively compared to initiation at three months postoperatively. The results revealed that the VSS score was significantly lower in the former group, with a difference of 1.70 (95% CI, 1.33–2.08) (Fig. 5).

### Complications

No severe complications were reported in included studies. However, transient erythema or swelling following Er:YAG laser treatment is commonly observed [15], with sporadic reports in other types of laser therapy. Additionally, Mossaad et al. documented patients complaining of discomforting pain during the treatment [17]. Overall, laser therapy is considered a safe treatment for preventing and treating CL/P scars.

## Discussion

This study represents the first systematic review and meta-analysis of laser therapy for scar management following CL/P repair surgery. Through comprehensive analysis of nine original articles, our findings indicate that various types of laser treatments are effective in improving scars post-CL/P repair surgery, exhibiting excellent safety, with early intervention showing superior efficacy.

The objective of treating cleft lip scars is to prevent scar hypertrophy or contracture and correct secondary deformities [3]. Specific repair methods vary depending on the severity and extent of the defect. These methods range from surface adjustments to surgical interventions, which involve deeper layers, such as the orbicularis oris muscle. Laser therapy primarily targets surface repair, facilitating wound healing and preventing abnormal scar formation. The initial descriptions of laser scar treatment can be traced back to 1993 when Alster et al. first introduced the use of pulsed dye laser for treating erythematous and hypertrophic scars [18]. Then Er:YAG resurfacing lasers and full surface ablative CO_2_ lasers were developed, and the former was the first laser type applied in improving CL/P scar [15]. Innovations in fractional laser technology thereafter enhanced the safety of laser treatments and facilitated their application in scar management [19].

Different lasers target various biological components or process. PDL is a vascular laser, with its wavelength selectively absorbed by oxygenated hemoglobin [7]. The laser produces vascular injury and coagulates the microvasculature of the scar, thereby deprive nutrients and potentially impairing fibroblast proliferation. Chi et al. [11] treated CL/P scars with PDL two weeks postoperatively, when angiogenesis is extremely active, demonstrating significant improvement. Er: YAG (2,940 nm) and CO_2_ (10,600 nm) lasers are ablative resurfacing lasers, with their wavelengths selectively absorbed by water. These lasers heat the affected epidermal and dermal regions above 100 °C, vaporizing the target tissue. Er:YAG laser operates at a wavelength of 2,940 nm, showing a high absorption coefficient for water. This enables the ablation of thin tissue layers (5–20 μm) and minimizes residual heat damage, potentially resulting in pinpoint bleeding [7]. On the contrary, the CO2 laser exhibits weaker water absorption, causing the vaporization of thicker tissue layers (20–30 μm) with a residual thermal injury zone of 50–130 μm, generating a bloodless operative field. Thus, Er:YAG laser treatment usually accompanied with erythema or swelling but these symptoms are only sporadic in studies using CO_2_ fractional resurfacing technology. Fractional CO_2_ laser is the most common laser used in CL/P scar treatment based on our review. The laser can inhibit angiogenesis in the early stage and collagen production in the remodeling stage, thereby improve the scar formation [20]. Our results show the early intervention (within 1 month after surgery) is better than late intervention, in line with the 2020 consensus. Peng et al. [14] combined the intense pulsed light and CO_2_ laser; the former degrading the microvasculature and the latter stimulating collagen remodeling. As Peng's theory, IPL is administered monthly, starting two weeks postoperatively, to diminish scar vascularization. CO_2_ laser therapy initiates one month postoperatively and recurs every three months to enhance treatment efficacy. The experimental group showed a significantly low VSS score (0.16 ± 0.55) at the 6-month follow-up, indicating excellent outcomes with early combined intervention. Although different lasers are effective in CL/P treatment, the optimal treatment pattern still need further exploration: Is combination of different lasers better than just one laser? What is the best laser type for early intervention of CL/P scar?

Currently, the assessment of scar severity primarily relies on evaluating texture, color, size, and other attributes [4]. Assessment tools include both subjective and objective evaluation methods. The most commonly used subjective assessment tool is the VSS, which includes four subdomains: pigmentation, vascularity, pliability, and height [21]. It employs a semi-quantitative method to assess scars, with scores ranging from 0 to 13, where 0 represents normal and 13 represents severe scar. PSAQ was developed in 2009 [22], comprising four dimensions: appearance, consciousness, satisfaction with appearance, and satisfaction with symptoms. This scale has been subsequently modified and applied in later studies [11, 16]. Objective assessment of scars involves techniques such as ultrasonographic measurement of scar thickness [9] and photographic measurement of scar area [11]. However, there is currently no widely accepted comprehensive objective scar assessment tool.

This systematic review and meta-analysis have several limitations. Firstly, the outcome indicators and data presentation varied in different studies which posed challenges in uniformly interpreting the results. Secondly, most of included studies only had follow-up periods ranging from 6 to 12 months, necessitating longer follow-up durations to explore long-term effects and detect recurrence. Thirdly, the number of high-quality randomized controlled trials included was quite limited. We anticipate more high-quality studies in the future to compare the therapeutic effects of different laser therapies and assess the advantages and disadvantages of various laser types.

## Conclusion

This meta-analysis supports the use of laser therapy for aesthetically improving scars following CL/P repair surgery, while also proving its sufficient safety. The effects of laser therapy on CL/P scars, as evaluated by other indicators, such as parameters of scar size and some other subjective rating scales, have also been comprehensively described. Notably, early intervention within one month postoperatively is more beneficial. Further high-quality RCTs are expected to explore the optimal laser therapy pattern in the future.

## Supplementary information

Below is the link to the electronic supplementary material.

Supplemental Figure 1 Funnel plot of included studies (PNG 61.7 KB)

High Resolution Image (EPS 68.4 KB)

## Data Availability

The data in this manuscript are extracted from the published primary studies, all of which are duly referenced within the text and fully listed in the reference section. The data extracted are summarized in the tables, figures, and supplementary material within the manuscript. Original detailed excel sheets can be obtained from the corresponding authors if requested.
