# Supplementary figures and images for: Laser therapy for treating cleft lip or/and palate scarring—a systematic review and meta-analysis
Source: Lasers Med Sci. 2024 Jun 20;39(1):160. doi: 10.1007/s10103-024-04082-3 (PMC11189991; doi:10.1007/s10103-024-04082-3)

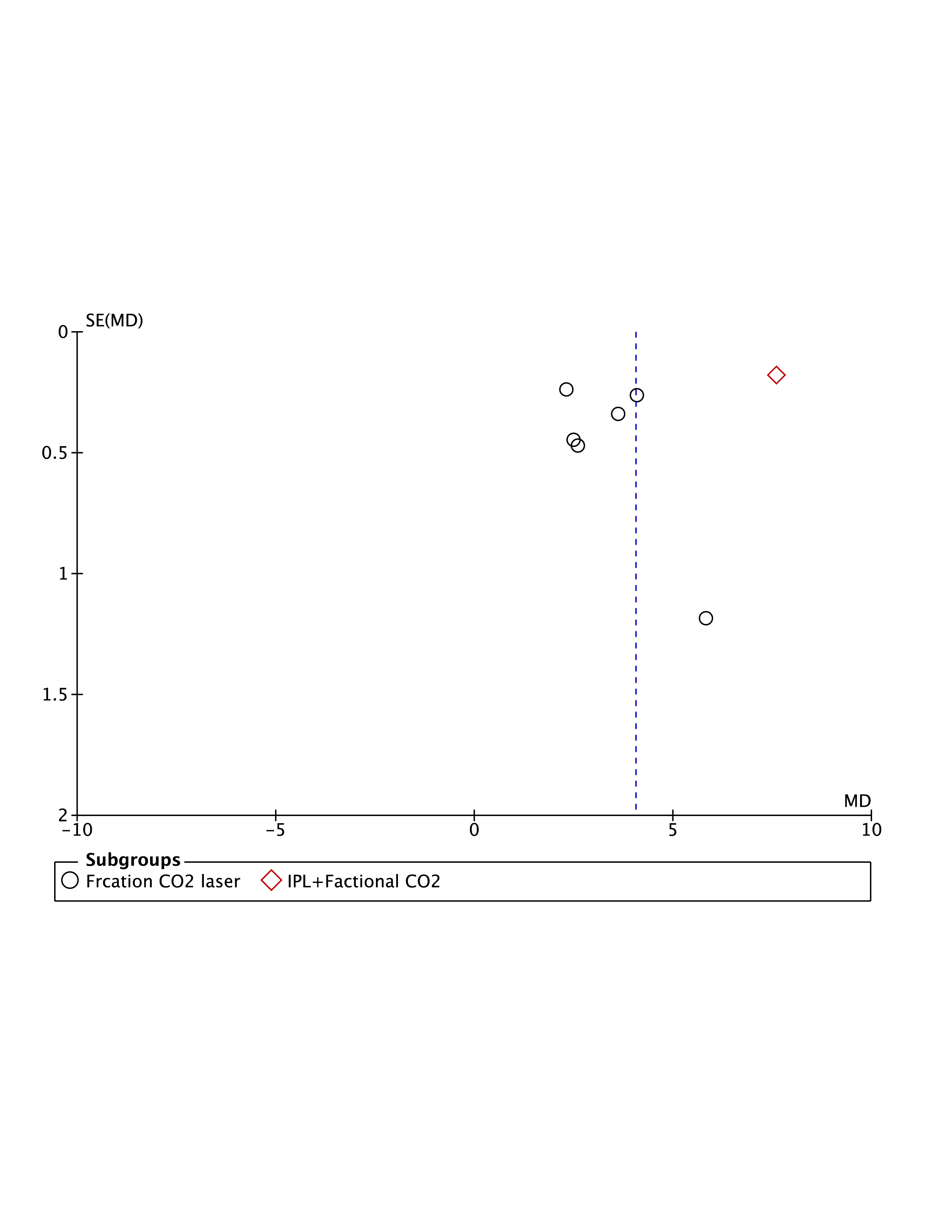

Supplement: Supplementary file 1 — Supplemental Figure 1 Funnel plot of included studies (PNG 61.7 KB) [file 10103_2024_4082_Fig6_ESM.png]
